# Imputation method for lifetime exposure assessment in air pollution epidemiologic studies

**DOI:** 10.1186/1476-069X-12-62

**Published:** 2013-08-07

**Authors:** Jan Beyea, Steven D Stellman, Susan Teitelbaum, Irina Mordukhovich, Marilie D Gammon

**Affiliations:** 1 Consulting in the Public Interest (CIPI), Lambertville, NJ; 2 Department of Epidemiology, Mailman School of Public Health, Columbia University, New York, NY, USA; 3 Department of Preventive Medicine, Mount Sinai School of Medicine, New York, NY, USA; 4 Department of Epidemiology, University of North Carolina at Chapel Hill, Raleigh, North Carolina, USA

**Keywords:** Exposure, Air pollution, Traffic, Benzo(a)pyrene, PAH, Multiple imputation, Epidemiology, In-migration, Dose

## Abstract

**Background:**

Environmental epidemiology, when focused on the life course of exposure to a specific pollutant, requires historical exposure estimates that are difficult to obtain for the full time period due to gaps in the historical record, especially in earlier years. We show that these gaps can be filled by applying multiple imputation methods to a formal risk equation that incorporates lifetime exposure. We also address challenges that arise, including choice of imputation method, potential bias in regression coefficients, and uncertainty in age-at-exposure sensitivities.

**Methods:**

During time periods when parameters needed in the risk equation are missing for an individual, the parameters are filled by an imputation model using group level information or interpolation. A random component is added to match the variance found in the estimates for study subjects not needing imputation. The process is repeated to obtain multiple data sets, whose regressions against health data can be combined statistically to develop confidence limits using Rubin’s rules to account for the uncertainty introduced by the imputations. To test for possible recall bias between cases and controls, which can occur when historical residence location is obtained by interview, and which can lead to misclassification of imputed exposure by disease status, we introduce an “incompleteness index,” equal to the percentage of dose imputed (PDI) for a subject. “Effective doses” can be computed using different functional dependencies of relative risk on age of exposure, allowing intercomparison of different risk models. To illustrate our approach, we quantify lifetime exposure (dose) from traffic air pollution in an established case–control study on Long Island, New York, where considerable in-migration occurred over a period of many decades.

**Results:**

The major result is the described approach to imputation. The illustrative example revealed potential recall bias, suggesting that regressions against health data should be done as a function of PDI to check for consistency of results. The 1% of study subjects who lived for long durations near heavily trafficked intersections, had very high cumulative exposures. Thus, imputation methods must be designed to reproduce non-standard distributions.

**Conclusions:**

Our approach meets a number of methodological challenges to extending historical exposure reconstruction over a lifetime and shows promise for environmental epidemiology. Application to assessment of breast cancer risks will be reported in a subsequent manuscript.

## Background

Historical reconstruction of exposure offers the opportunity to cover periods of a person's life when they may be most vulnerable [1,2], but data challenges may make it difficult to attempt such a reconstruction for an epidemiologic study. Despite these difficulties, long-term assessment of residential exposure to pollutants is increasingly being used in environmental epidemiology of disease, particularly cancer [3-5]. As part of an effort in a large-scale study of environmental exposures and breast cancer [6], we wanted to add individualized inhalation exposures of PAH from traffic emissions to a previously collected set of individualized PAH exposures from diet and active/passive smoking. Within the category of PAH air exposure, we focused on traffic emissions because they are a major source of both indoor and outdoor exposures to PAH, and often the largest source in areas near cities, as has been confirmed in a number of experimental studies [7-12]. However, it was necessary to confront a number of methodological challenges before inhalation exposure from traffic could be obtained over a lifetime and before it could be totaled appropriately.

First, it was necessary to decide how to combine yearly exposures over decades. There was no guarantee that simply adding them to obtain a dose would capture possible differences in relative risk per unit of exposure at different study-subject ages. We approached this challenge by computing “effective doses” assuming a range of different functional dependencies on age of exposure. Effective doses are computed by summing functions of yearly exposure. Having a suite of effective doses available for use in regressions against health outcomes allows for broad coverage of the possible age-sensitivities.

Second, it was necessary to deal with exposure before study subjects arrived in the study area, as well as exposure that occurred in years before desirable records on emissions and traffic flows were available. A third challenge arose, because the earlier in time we went back to estimate exposure, the greater the percentage we found of missing residential information. To deal with these two types of missing-data problems we propose the use of multiple imputation, namely constructing a series of data sets with missing parameters randomly drawn from a set of predetermined values derived from an imputation model. The resulting multiple data sets are then available to be regressed against health outcomes. On the positive side of this approach, random errors in regression coefficients are reduced within an imputation data set, because sample sizes are increased. On the negative side, there is new variance in regression coefficients introduced when comparing results between imputed data sets. The net effect of these competing tendencies on overall variance can be assessed using Rubin’s Rules or alternate formulations [13-15]. With this combined variance in hand, confidence limits on regression coefficients can be obtained that explicitly include uncertainty introduced by imputation of missing values.

The fourth challenge we faced was a consequence of limitations in the imputation process itself, namely the vulnerability to increased risk of recall bias, when information needed to impute a missing dose component is based in part on questionnaire answers obtained from individual study participants. This can lead to bias in regression coefficients and, therefore, the possibility of finding false or hidden associations with health outcome.

The quantity imputed is the portion of the total dose accumulated during periods of missing location data or missing emissions data. The percentage of the missing component can vary in magnitude by study subject. Although dealing with partially missing information presented a challenge, it also offered an opportunity to check the robustness of regressions with imputed data, and thus test for recall bias. To this end, we introduce the “percentage of dose imputed” (PDI) as a continuous index that allows limiting analysis to groups of study subjects depending on their degree of imputation. With PDI limit set to 0, the analysis is a complete-case analysis. With the limit on PDI set to 100%, the full population is analyzed. In this way, regression of health data could be performed as a function of the limit on PDI to see if the results were consistent as larger and larger components of imputed dose were allowed for study subjects. If there was consistency, then it would be reasonable to extend the results of a complete-case analysis to larger segments of the full population.

A fifth methodological challenge arose, because the observed doses among the subjects with complete information turned out to be right skewed: specifically in our case–control study the 1% of women who had lived for a long time near heavily trafficked intersections. This presented a challenge to our imputing doses in such a way as to properly populate “outlier” values. To deal with this type of imputation challenge, we propose approaches that do not assume standard distributions; moreover we recommend using more than one approach to allow testing the sensitivity of the regression results to the choice of imputation model.

All of these challenges are likely to be faced, to a lesser or greater degree, when researchers contemplate studies that require individualized historical exposures of adults over a lifetime, or even a great portion of it. As a result of all the potential difficulties, historical exposure is often ignored and analysts look for associations with exposures in recent periods when data are complete. However, doing so can introduce exposure misclassification, depending on the historical mobility of the population and the variation of exposure in place and time, with a resulting tendency to bias results towards the null. Associations can be missed [16]; and periods of greatest importance for health effects go unnoticed [4]. One approach to dealing with missing historical exposure data is to restrict analysis to a subset of the study population with complete data, but such a restriction can decrease sample size drastically and raises questions about applicability of results to the entire study population. Thus, it was of interest to explore new ways to accomplish multiple imputation in the historical context and to find ways to test for recall bias.

We illustrate the approaches developed to deal with the challenges using data from a population-based, case control study of breast cancer on Long Island, New York, a cancer for which a life-course approach to research is strongly recommended [17].

## Methods

### Risk equation

Our ultimate goal is to obtain dose estimates that can be used as predictor variables in logistic regression models of disease risk in which log (odds of disease) is linear in integrated exposure opportunity (dose).^a^

(1) $$\ln\left(\frac{p_{i}}{1-p_{i}}\right)=A+\beta *Z_{\mathrm{calc}}\left(i,y\right)+Z_{\mathrm{imp}}\left(i,y\right)$$

In Eq. 1, *p*_*i*_ is the probability of an individual, *i*, becoming a case in year, *y*. β is a regression coefficient. *Z*_calc_(*i*, *y*) is the exposure opportunity accumulated for time periods when information is complete for individuals, i, permitting individualized estimates. *Z*_imp_(*i*, *y*), is an imputed dose for any time periods of interest when information needed for an individualized exposure estimate is lacking. Only the missing subcomponents of the exposure model are imputed, so that as much physical and biologic knowledge is retained. *Z*_imp_(*i*, *y*), by definition, will be zero for the set of subjects included in complete-case analysis.

Covariates that represent known risk factors for the disease under study, as well as possible confounding variables, are implicitly included in A. The magnitude of *Z*_calc_(*i*, *y*) + *Z*_imp_(*i*, *y*) determines the risk that subject, *i*, will become a case in year, *y*, arising from exposure terminating in a given year, such as a year related to a biological state (e.g., menopause), or at the end of the study period, which is the value chosen for our numerical examples.

To account for different sensitivity to age of exposure, the *Z*(*i*, *y*) terms in Equation 2 allow for more than a simple sum over short-term exposure calculations. Within the sum, each short-term calculation is multiplied by a “biological effectiveness” factor potentially dependent on age at exposure and age of disease onset.

(2) $$Z\left(i,y\right)=\sum_{y'=y_{b}}^{y_{\mathrm{stop}}}E\left(y',i\right)*B\left(y'-y_{b},y-y_{b}\right),$$

*E*(*y’, i*) is the short-term exposure at date, *y′*. The variable, *y*, is the year of disease diagnosis. The sum in Eq. 2 is over the dates, *y′*, running from birth year, *y*_b_, to the last date of exposure interest, *y*_stop_. *B*(*y′-y*_b_*, y-y*_b_) is a relative risk that compares the effect of unit exposure at age, *y'-y*_b_, to the effect at a standard age of exposure, say 1-years old. Thus, if a unit dose at age 10 will double the risk of disease caused by a unit dose at age 1, the *B*-variable for age 10 equals 2. In this way, the *Z*-terms, which depend on *B*, can be called effective doses.

Subjects with the same *Z*-dose have the same increase in log (odds of disease) compared to an unexposed person, no matter how their exposure was distributed over time. Rather than define the baseline of *B* as a single exposure in a particular year, the baseline can be defined relative to a standard distribution of exposure over a time period. To include birth-cohort effects, for instance in a cohort study, the *B*-factor would include a separate entry for birth year, *y*_b_, and be written as *B*(*y′-y*_b_*, y-y*_b_*, y*_b_).

In this paper, we consider four functional forms for *B*, distinguished by subscripts 1 through 4, which span a wide range of possible age sensitivities:

*B*_1_: *B* = Constant;

*B*_2_: *B* is inversely proportional to square of age of onset, which increases relative risk at young ages (as in best mathematical fit to data for relative risk of breast cancer following ionizing radiation exposure [2]);

*B*_3_: *B* vanishes except at peak historical exposure (threshold model). (To work as a threshold model, *B*_*3*_ can be used in conjunction with a threshold step function added to the risk equation.);

*B*_4_: *B* is negligible except in the last *k*-years of the study (pure promoter model).

We note that a latency period can be implemented in any of the above models simply by changing the stop year in the summations.

The equation for effective dose, Eq. 2, involves a summation over a study subject’s entire life, but individual values of each variable that are needed to compute the sum are not usually completely available since birth. To fill in the missing information, we replace variables at various times with group level values. The time steps over which emissions must be tracked in equation 2 depends on the model being used to assign individualized exposure estimates. The base unit might be months, or any duration corresponding to data typically available depending on disease and the nature of the short-term exposure model. For instance, the natural starting unit might be hours for a meteorological dispersion model that tracks emissions that vary by hour of day and accounts for the correlated meteorological conditions that also change hour by hour, such as rainfall, wind speed, and wind direction. Such an hourly model may be testable against hourly air concentration data collected at monitoring stations [18,19]. As long as average emissions per month do not change rapidly, it possible to do the hourly summations once for each month of the year and use the results for the same month in each year without much loss in accuracy. Data averaged by month is appropriate when movement of study subjects between residences is known by month of the year.

It is useful to separate the exposure, *E*(*y′*, i), in Eq. 2 into sums or products of terms that reflect the underlying model of exposure, so that terms needing imputation can be separately identified. For a land use regression model the natural separation might be a linear combination of terms, including air concentrations at monitoring stations [20]. For a meteorological dispersion model, it would be natural to break *E*(*y'*, i) into a product of terms. Regardless of whether the separation is a sum or product, whenever there is a time that a particular term or subpart is missing for an individual, it will need to be imputed according to an imputation model. In either case, random numbers are used in the multiple imputation model as part of the process of drawing parameter values from a predetermined set of options.

For the traffic emission example, we assume organ doses are linearly related to air concentrations and expand Eq. 2 as a product that reflects the underlying physical process of tailpipe carcinogens being emitted from vehicles and those carcinogens being transported through the air to reach a residence:

(3) $$E\left(y',i\right)=\epsilon \left(y'\right)*T\left(y'\right)*D\left(y',r_{i}\left\lfloor y'\right\rfloor \right),$$

ϵ(*y′*) in Eq. 3 is the average emission per km from the tailpipe of an average vehicle along the road network at a particular date, *y′*, determined from average US values [21]. *T*(*y′*), in Eq. 3, is the average number of vehicles per km on an average road in the modeling area in the year, *y′*. Since 1970 it has increased by a factor of 2.7 in the entire U.S. [21]. *D*(*y′, r*_*i*_[*y*]), in Eq. 3, is the “dispersion function,” also called the “transfer function” [22]. It relates emissions from the entire road network to exposure at individual residences, *r*_*i*_. It is computed by tracking puffs of pollution as they change direction with hourly-wind changes, as they expand at rates dependent on meteorological conditions, as their concentration declines due to depletion processes, and as they finally arrive at residences.

*D* represents one hour’s exposure received at the residence of a subject, *r*_*i*_[*y′*] at a date, *y′*, from one unit of pollutant emitted from all the vehicles on the road network, some of whose emissions may have started their airborne travel several hours earlier. To complete the calculation of *D*, the received exposure is divided by the average number of cars per km on the roadway, *T*(*y′*). In this paper, any imputation of Eq. 3 that is needed for a study subject in year, *y′*, is done on the transfer function, *D*(*y′*), as long as data for ϵ() and *T*() are available. *D*-values tend to be roughly constant over historical time, changing only as the relative traffic pattern changes in different parts of the modeling region. In addition to depending on the road network, *D*-values depend on region-specific meteorological factors that enter the transport dispersion model.

### Incompleteness index

As previously stated, due to incompleteness in reported residence addresses and gaps in available historical exposure data, most epidemiologic studies cannot be expected to have a complete, individualized lifetime dose for every subject. We wish to assess the potential impact on estimates of associations between exposure and effect measures of filling these gaps with imputed values. To this end we define in Equation 4 an, “incompleteness index,” as the group-level imputed dose expressed as a percentage of the total dose:

(4) $$\begin{array}{l} \mathrm{Incompleteness}=\mathrm{PDI}\\ \;=\frac{100\%*\left(\text{imputed dose component}\right)}{\text{imputed dose component}+\text{modeled dose component}}\\ \;=\frac{100*Z_{\text{imp}}}{\left(Z_{\text{imp}}+Z_{\text{calc}}\right)}, \end{array}$$

Our approach is to use this incompleteness index (PDI) to define subsets of participants with varying contributions of group level components. Thus, we might compare regression or correlation results for a subset of study subjects with PDI’s = 0 to larger subsets with PDI's less than 20, 30, 40%, and so on. If the results are robust, with means and confidence limits that suggest a similar association, we can have confidence that imputation is not introducing artificial findings. Extrapolation of the results to the full sample of study subjects would become reasonable. Chi-square analysis of Tables of cases and controls grouped by intervals of PDI is a simple way to test for differential bias in imputation percentage.

It would also be reasonable to define an alternate incompleteness index based on the percentage of time that imputation was needed for a subject. However, this “percentage of time imputed” (PTI) is not as conservative an index as the percentage of dose imputed, particularly in situations where historical exposures varied greatly over time. A major advantage of setting a numerical value of PDI as a limit is that it insures that all study subjects have their degree of imputation constrained. In any case, PTI should be computed along with the PDI as a sensitivity check.

### Imputation of gaps in an individual’s dose

For the type of study we are considering, there are boundaries in space and time separating regions where individualized exposure estimates can be made. Yet, some exposure is likely to have occurred outside these boundaries, necessitating group level surrogates for these regions. Surrogates should be consistent at the boundaries such that averages and variances of exposure match the comparable quantities for subjects with individualized exposure. For instance, consider a situation where reliable local traffic and emission data were not available before 1960. Suppose that the number of registered vehicles in a state is taken as the group level surrogate for exposure prior to 1960. For consistency, when the surrogate is extended to a year when it is not needed, e.g., 1960, it should have the same average as the individualized exposures that were computed for 1960. This consistency can be accomplished by scaling the surrogate by the average individualized exposure in 1960, *dose*(1960), and dividing by the vehicle registration number in 1960, as in Equation 5.

(5) $$\mathrm{EarlySurrogate}\left(t,i\right)=V_{1}\left(i\right)*{\left(\frac{\mathrm{Vehicles}\left(t\right)}{\mathrm{Vehicles}\left(1960\right)}\right)}^{k_{1}}\mathrm{dose}\left(1960\right),$$

To individualize the surrogate in Eq. 5 for study subject, *i*, the surrogate has been multiplied by a random variable, *V*_1_(*i*), which is uniquely chosen for each study subject in each imputed data set. The distribution of *V*_1_ should be chosen to have a mean of unity and a variance that matches the variance in 1960 of the individualized exposure estimates. For purposes of allowing sensitivity analysis, we have added a parameter, *k*_1_, as an exponent in Eq. 5, which could be varied around unity, should one want to test regressions with health data for different assumptions about the relationship between early vehicle registration numbers and traffic exposure. Standardization of the surrogate is carried out separately for cases and controls. Although Eq. 5 is independent of geographic locations of residences, it would also be possible to perform the analysis separately by geographical subregions of the study area.

A surrogate like the one defined in Equation 5 is the very opposite of an individualized dose, which implies considerable possible misclassification. And, the surrogate is likely to be highly correlated with age, requiring careful control of the age-variable to avoid residual confounding. On the other hand, if exposure is particularly important at young ages, then exposures in the period requiring surrogates is likely to be important. Also, it is quite possible that increased cancer incidence with age is due in part to the longer time older people have to accumulate exposure and to develop cancer [23]. A middle ground position would be to compare regression coefficients with and without the early dose surrogate included. When included, the implicit assumption is that early dose effects are so strong biologically that an exposure estimate grossly misclassified at the individual level will still be adequate to improve the overall dose classification, thereby facilitating the detection of any effect. Excluding the early dose surrogate is equivalent to assuming a biologic model that has cancer risk from the exposure falling off in importance years after exposure.

The early surrogate cannot contribute directly to any recall bias, because no information from interview plays a role in its initial calculation. Recall bias could nevertheless carry over to the early surrogate, however, because the scaling factor which is computed separately by cases and controls is based on post-1960 individualized data that depends on subject recall of address locations. For some of the numerical examples given in this paper, we exclude the early dose surrogate, because including it does not help us elucidate potential recall bias using the PDI, which is a major focus of the paper.

It may not always be possible to find a boundary on which to match a surrogate. This is the case for subjects during periods when they resided within the temporal boundaries of the study, but outside the spatial regions. The natural way to generate dose surrogates for these subjects during their out-of-area periods is to retain as much data needed for individual calculations as applies outside the spatial region, but use average values for non-available parameters. The needed average values can be computed by summing over the individualized values calculable for study subjects inside the spatial study area during the same time period.

There is no a-priori reason for expecting the averages to be the same in and outside the region, so we multiply the results by a common sensitivity parameter, *k*_2_, which can be varied in regressions with health data to check the importance of the default assumption that the averages are the same. Let, *F(t, p*_1_*, p*_2_*, p*_3_*, p*_4_*)*, be an individualized exposure at time, *t*, for a subject residing inside the study area, given values of the parameters, *p*_1_, *p*_2_, *p*_3_, and *p*_4_. Assume that *p*_3_ and *p*_4_ are unknown for study subjects outside of the area. Then, the surrogate for a time when a subject, *i*, is out of the study area, becomes:

(6) $$\begin{array}{l} \text{OutOfAreaSurrgate}\left(t,i\right)=k_{2}*V_{2}\left(t,i\right)\\ \;\;*F\left(t,p_{1},p_{2},<p_{3}>,<p_{4}>\right), \end{array}$$

where *p*_3_ and *p*_4_ have been replaced by < *p*_3_ > and < *p*_4_>, averages over values for other study subjects, namely those subjects residing in the study area at time, *t*. Once again, individualization can be carried out by multiplying by a random variable with mean unity, which we call, *V*_2_. The variance of *V*_2_ would be chosen to match the variance of subjects with individualized doses inside the study area. With this surrogate, the variance, *V*_*2*_, can change over time, so that matching needs to be carried out more than once, say, every five years. As with the earlier surrogate, standardization is done separately for cases and controls.

We note that the parameter, *k*_2_, could also serve as a regression coefficient to be determined as part of the regression with health data. An alternate approach would be to individualize the parameters < *p*_3_ > and < p_*4*_ > separately. See supplementary information for a discussion of these options.

The two group-level surrogates defined above refer to dose accumulated over different time periods in a subject's life. Obtaining an individual's imputed dose requires summing over both, if both are non-zero.

### Imputation of missing residence information

There may be additional imputation required for a study subject, even within the temporal and spatial boundaries of the main study, for instance, to impute an individualized, *D*-value or other indicator of local exposure. In our example, historical street addresses, including house number, were obtained by interview, which meant relying on the memory of study subjects for input to geocoding software. Additional file 1: Figure S1-1 shows the percentage of geocoding success by year of arrival at residence. For 1960, the success rate was 65%; therefore, 35% of the addresses in that year needed imputation.

We consider two ways to carry out the imputation on the theory that having two methods to compare regression results is a safeguard. Consistent results have the potential to increase confidence in the outcome; inconsistent results would be a warning that the imputation approach was problematic.

Within the mathematical function that defines short-term exposure there will be some residence indicator, whether it be proximity to roads or a meteorological transfer function specific to a residence, such as the *D*-values discussed earlier. When a quantity like *D* is missing, the easiest method of imputation, suggested by Raaschou-Nielsen et al. [5] is to interpolate between values at residences before and after the missing value. To obtain values that differ between different imputed data sets, we can multiply the interpolated imputed exposure quantity by a random number with a log normal distribution whose variance is obtained from a lognormal fit to the two calculable quantities about which one is interpolating. For each imputed data set, a distinct value is randomly drawn from the derived lognormal distribution. Alternatively, when there is a missing quantity at starting or stopping dates, extrapolation can be used. In such a case, zero variance is assigned to the lognormal distribution and there is, therefore, no difference in interpolated dose component for a study subject in different imputed data sets for the missing residence period.

This method has the advantage that it relies solely on individual information and does not require the use of covariates in helping to choose the imputed dispersion coefficient. It has the disadvantage that, if there is a long tail in the exposure distribution, it can overstate the imputed exposure components when one of those high values is used for imputation.

Our second method relies on the fact that when information on residence location is obtained by questionnaire, it is likely that most residents will be able to remember at least the city, town, or village of the residence, even if they are not able to remember the full address. This means their location can be narrowed; in the US, we have found location could be narrowed to the Census Division Place level 80% of the time in our example study. Thus, it is possible to narrow the imputation to values of the observed data within the corresponding Place. In what follows, we call this, “imputation by place.” To handle the situation when a census division cannot be assigned or when the number of either cases or controls in a census Place is less than five (11% of locations in our example study), it is appropriate to fall back on the interpolation method.

With imputation by place, it is good practice to use covariates available for a subject, such as income or education, in picking the imputed values, because they could have influenced the area chosen for a residence. One of the limitations of multiple imputation is that there are situations involving missingness not at random that can introduce bias. This possibility can be reduced by including a large number of covariates in the imputation model, thereby increasing the chances that any variables predictive of missing values are incorporated [15].

Observed values of covariates included in the imputation-by-place algorithm for our breast cancer example were *D*, smoking status (passive and active), education, history of fertility problems, history of benign breast disease, family history of breast cancer, combined estrogen receptor and progesterone receptor status among cases, parity, BMI, dietary intake of PAH, lifetime intakes of grilled or barbecued and smoked meats, lifetime alcohol intake, and age at diagnosis. Case–control status was also included, which at first consideration seems circular; however, including it is a valid part of multiple imputation [24,25]. Otherwise, patterns in the observed data might not be transferred to the unobserved data during imputation.

In contrast to imputation by interpolation, which may overestimate high values of dose, when there is a highly right-skewed distribution, the method of imputation by place may overestimate some low values of dose, should the values in the top tail of the distribution be correlated with the density of road intersections, as would be expected. This contrasting tendency makes use of the two methods in regression of health outcomes a useful test of the robustness of results.

### Breast cancer example

#### Study population

We use data on 3,064 female breast cancer cases and controls who were residents of Long Island, New York, between August, 1996, and July, 1997 [26]. Cases were women newly diagnosed with a first primary *in situ* or invasive breast cancer; controls under 65 were identified through random digit dialing and by Health Care Finance Administration (HCFA) rosters for those 65 years of age and older. Controls were frequency matched to the expected age distribution of cases.

**Figure 1:**
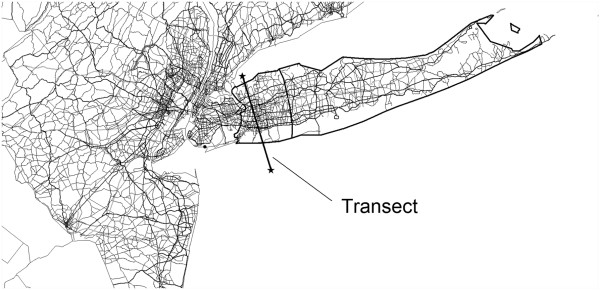
**Major roads from which emissions were tracked.** Study participants came from the area on the island marked off with bold boundaries, 150-km in length. The transect defines the location of the predicted air concentrations shown in Figure 2. Long Island Breast Cancer Study Project, 1996–1997 [26].

**Figure 2:**
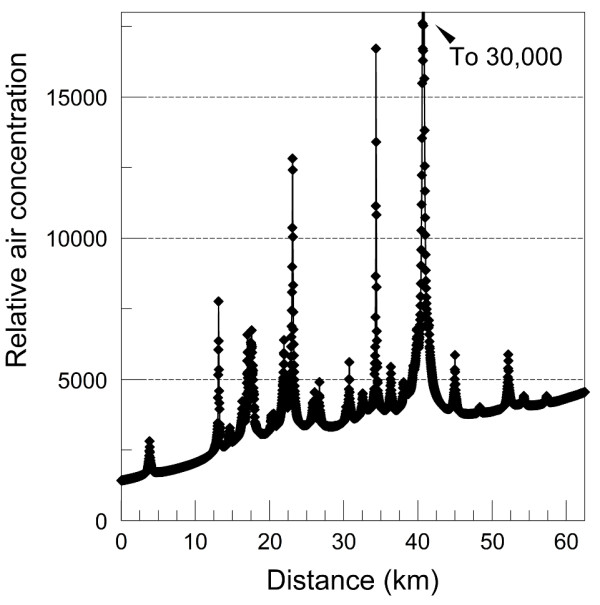
**Relative 1-hour air concentrations modeled for 1995 along a transect across Nassau County, Long Island.** The origin is at the ocean side of the transect shown in Figure 1. Long Island Breast Cancer Study Project, 1996–1997 [26].

Residence locations on Long Island, but not outside of Long Island, were identified at the time of the in-person, structured interview, starting with a subject’s earliest recollection. This resulted in a total of 8,319 locations in Nassau and Suffolk counties divided among the 3,064 women. Address coordinates at the street level were identifiable for 5,383 of these locations using commercial geocoding software (BLR Inc., now part of GDT, Lebanon, NH), with 95% of the remainder identifiable only to the 5-digit Census category, “Place,” due to incomplete address information. A census division, “Place,” consists of consolidated cities and incorporated places, such as town or village. Certain unincorporated areas may also be assigned a Place number http://www.census.gov/geo/www/codes/place/download.html. The average population of a study-area Place in the 1990 Census was approximately ten thousand, or about 1% of the total population of Long Island. Location to the Place level is sufficient to capture the urbanization gradient along the length of the elongated Island study area [18]. For consistency, the street maps used for geocoding were used to model dispersion of traffic pollution. The study area and surrounding traffic network are shown in Figure 1. Relative 1-hour air concentrations predicted by the geographic dispersion model in 1995 along a transect across Long Island are shown in Figure 2.

Benzo(a)pyrene (BaP) emitted from vehicle tailpipes was the pollutant proxy analyzed for the numerical examples, because there is an abundance of good historical data [21]. The emission per km variable, ϵ(y’), in Eq. 3 peaked in the US in the mid-1970s, declining rapidly with the introduction of catalytic converters until the late 1980s [21]. This means that per unit of time, exposure in the pre-1980 period was likely to be much higher than after 1985, adding large temporal variation to the spatial variation associated with proximity to traffic evident in Figure 2.

Data for BaP and other PAH tailpipe emissions extend back only to 1960, which sets that year as a boundary for shifting to group level information. In the absence of information, and no engineering reason evident in the literature to think otherwise, we took ϵ(*y′*) to be a constant prior to 1960 equal to the known value in 1960. We also took the dispersion variable, *D*, as constant, equal to the average value computed in 1960 for residences in the study area. We used vehicle registrations in New York State, for which date existed back to 1900, as the surrogate for traffic flow rates for the pre-1960 period, as in Eq. 5. See Additional file 1: Figure S1-2

#### The LIBCSP meteorological dispersion model

The LIBCSP meteorological dispersion model was developed specifically for the Long Island Breast Cancer Study Project's analysis of traffic exposures, although it has been adapted for use in the Buffalo, New York area, with separate tests carried out in that region [19,27]. A special feature of the model is its allowance for separate emissions from road intersections, which are known to be higher than during cruise emissions [28,29]. The model does not account for street canyon effects, but that is not a necessity for Long Island.

The contribution from each road segment to the air concentration at a downwind receptor residence was computed within 100 meters of a road using a highway line-source model [30] applied to each of the 500,000, straight-line road segments in the traffic network. The “Chock” highway model was chosen because it gave the best fit, when compared to a suite of models tested, to tracer concentrations near the Long Island Expressway as part of a test carried out by the New York Department of Environmental Protection [31]. R^2^ values ranged from 0.75 to 0.92 for various meteorological conditions and angles to the road.

Beyond 100 meters, we used a standard Gaussian puff dispersion model (equivalent to the USEPA’s “RAM“ model [32]). Additional file 2: Table S2-3 describes the default parameters and data sources used in the dispersion model. Further details can be found in the text of Additional file 2, including an explanation of the approach used to shift between different meteorological models at 100 meters and a description of how we account for background BaP blown into the study area from outside it.

Total concentration at a study subject’s residence was computed as the sum of the contributions from the approximately 500,000 source segments. This concentration at a residence forms the basis for the transfer function, *D*.

The transport model used to compute the transfer function, *D*, was previously validated against BaP soil measurements at approximately 500 residences [18]. A key result was higher than average emissions coming from traffic intersections, which were modeled separately. See Additional file 1: Figure S1-3 for a graphic showing separately-modeled intersection regions superimposed on a segment of the road network.

### Imputation to account for missing residence location in the Long Island example

**Figure 3:**
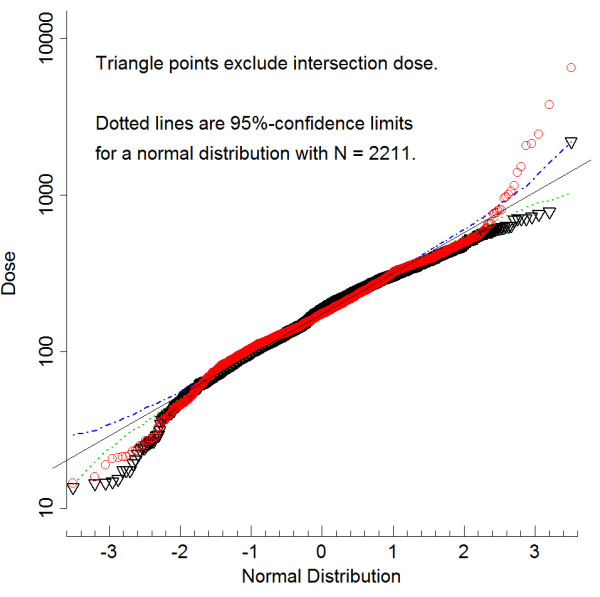
**Normal probability plots of cumulative dose from 1960 through 1995.** With and without intersection dose, both with percentage of dose imputed (PDI) < = 90%, N = 2211. 95% confidence limits shown. Relative dose units: 1 unit = 1 year's average dose in 1995. Long Island Breast Cancer Study Project, 1996–1997 [26].

Imputation is needed during times when subjects reside in the study area, but their residence location is not known with enough precision to determine accurate latitudes and longitudes, quantities that are necessary to obtain individualized exposure. In the example data presented in this paper, the primary method of imputation used was imputation by place. We imputed a *D*-value from the set of *D*’s calculated for other women residing in the same census division Place. The choice of imputed values was conditioned on the observed covariates discussed earlier using fully conditional specification as implemented in the R-program, “Multivariate Imputation by Chained Equations (MICE)” [33]. MICE is but one of a number of imputation programs available in statistics computer packages. Fifteen different sets of data were calculated with missing transfer functions and any missing covariates imputed. For further details of the example imputation, see text in Additional file 2. The input to the imputation program is a matrix file with "NA"s for missing transfer functions and missing covariates; the output is a set of files with the NAs replaced with numeric values, which generally differ from set to set. Because of the long-tailed distribution found in exploratory analysis (see Figure 3), there was no standard distribution, e.g., lognormal, that could be used to fill the missing transfer functions. Therefore, we ran the MICE program with the option to sample randomly from *D*-values fully calculable for other subjects. These samplings are not completely random, however; the program uses regression techniques to weight the choices according to any tendency of different transfer functions to cluster by covariates. The output files are then available for regression against health data and the variance of the results between imputed data sets plays a role in determining confidence limits using standard rules [14].

## Results

The main results from this paper are Eqs. 2, 3, 4, 5, 6 and the approaches we described to operationalize them. In this section, we discuss results from the Long Island example.

Different biological effectiveness functions: For the illustrative example, Pearson correlation coefficients between dose calculated with different biological effectiveness factors ranged from 0.33 to 0.79 with no limits set on PDI (PDI < = 100%). The range was 0.59 to 0.99 for complete-case analysis (PDI = 0). See Table 1 and Additional file 1: Tables S1-1 and S1-2.

**Table 1:** **Pearson correlation coefficients between doses computed with different functional forms for the biologic effectiveness factor, averaged over 15 imputations**^**a**^

**Samples of women limited by allowed percentage of imputed dose (PDI)**							
**Dose surrogate**	**CCA (PDI = 0%)**		**PDI < 20%**	**PDI < 40%**	**PDI < 60%**	**PDI < 80%**	**(PDI < = 100%)**
	**N:**	**547**	**1164**	**1477**	**1764**	**2051**	**2986**
Cumulative dose 1960-1995		(comparison dose variable)					
Dose for 1995 only (Promoter model)		0.59 (0.11)	0.39 (0.15)	0.39 (0.16)	0.39 (0.16)	0.41 (0.16)	0.33 (0.15)
Peak annual dose in 1960–1995 (Threshold model)		0.99 (0.00)	0.93 (0.00)	0.93 (0.00)	0.92 (0.01)	0.91 (0.01)	0.75 (0.017)
Cumulative dose X (onset age)^-2^ (Age sensitive model)^b^		0.89 (0.024)	0.87 (0.028)	0.85 (0.033)	0.84 (0.039)	0.82 (0.044)	0.79 (0.06)
Pre-1960 surrogate^c^		−0.020	−0.031	−0.031	−0.030	−0.016	+0.00044

^a^Numbers in parenthesis are the square root of the variance across imputations divided by the square root of 15, which provides an estimate of the standard deviation of the average over imputations.

^b^Weighting based on the mathematical fit to radiation risks of excess breast cancer in atomic bomb survivors.

^c^Calculated for one imputation only.

### Early surrogate dosage (before 1960)

The early surrogate dosage for the breast cancer study subjects averaged 50% of the post-1960 dose (calculated for the *B*_1_, constant sensitivity biologic model). Additional file 1: Figure S1-4 graphs the average dependency on age. The Pearson correlation coefficient was 0.6.

There was essentially zero correlation between the early surrogate and the cumulative dose post-1960. The Pearson correlation coefficient ranged from −0.05 to 0.001 as the limit on PDI ranged from 0 to 100%. This low correlation means that inclusion of the early surrogate in the dose summation could alter the distribution curve for total dose.

### Doses calculated after 1960 (*Z*_calc_ and *Z*_imp_)

**Figure 4:**
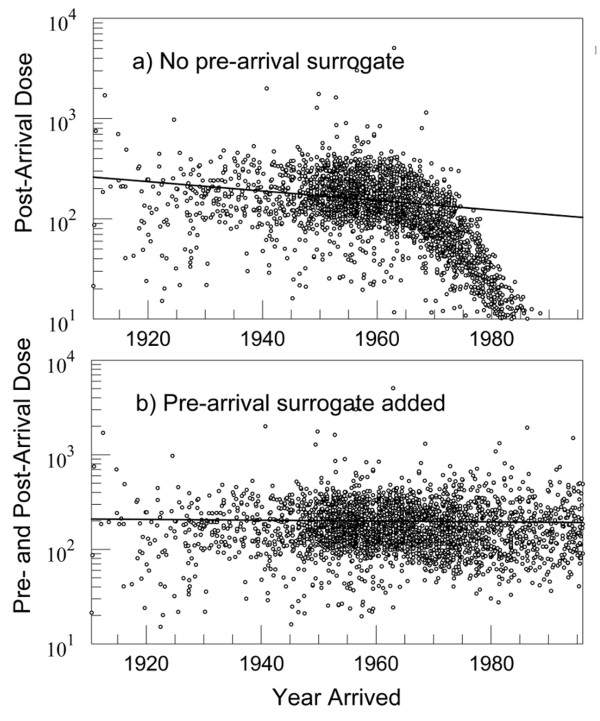
**Dose as a function of year of arrival in study area, with/without pre-arrival surrogate.** Relative dose units: 1 unit = 1 year’s average dose in 1995. Long Island Breast Cancer Study Project, 1996–1997 [26].

Of the 2,986 study subjects who arrived in the study area before 1996, about 55% of them arrived after 1960. Thus, individually modeled doses in the post-arrival period (*Z*_calc_) are strongly affected by arrival date because late arrivers are missing their out-of-area contribution. Pearson correlation coefficient with year of arrival = −0.37. Figure 4a graphs the relationship, showing how the *Z*_calc_, post-arrival doses fall off for late arriving study subjects. Without accounting for out-of-area exposure through imputation, i.e., by adding the *Z*_imp_ contribution, false differences in dose calculations can arise, should cases and controls, because of unmeasured covariates, have a different distribution of arrival times.

The *Z*_imp_ surrogate itself, like *Z*_calc_, is highly correlated with year of arrival, albeit with a different sign (Pearson coefficient = 0.56). Figure 4b shows how adding the post-arrival, out-of-area surrogate, *Z*_imp_, to the individualized dose component, *Z*_calc_, removes the dependence on year of arrival.

To avoid interpreting BaP as more than a PAH proxy [34], doses are presented in the Figures, not in units of inhaled milligrams of BaP, but in units of the 1995 average dose computed for women in the study area. Note that Figures 4a and 4b show graphs for but one imputed data set; however, the patterns are virtually identical at this level of detail for 14 additional imputed data sets, as is to be expected given the great variance around the mean dose curve (results not shown). Also note that a dose value in Figure 4b may be the sum of three terms, an out-of-area imputation, an in-area imputation, and a geographically modeled component.

Although adding *Z*_calc_ and *Z*_imp_ together eliminates the obvious way that recall bias could enter the estimation of doses, it is still necessary to go further and look for residual bias in *Z*_calc_ + *Z*_imp_, which can be done using the PDI variable. We performed a chi-square analysis of cases and controls grouped by intervals of PDI. The results are shown in Table 2. The p-value for rejecting the null hypothesis was 0.13, which is low enough to suggest some association between PDI and case status in our illustrative example, and therefore the possibility of differential bias.

**Table 2:** **Distribution of women by limits on incompleteness index (PDI**^**a**^**) in cumulative dose for one imputed data set**^**b**^**(Women who arrived before 1996)**

**Limits on PDI**^**a**^	**Number of control women**	**Number of case women**	**Total**
Zero (complete coverage)	267	280	547
>0 and < 20%	336	281	617
20% to < 40%	164	149	313
40% to < 60%	150	137	287
60% to < 80%	136	151	287
80% to < = 100%	465	470	935
Total	1518	1468	2986
Probability level for Chi-Square			0.13

^a^PDI = Percent of dose imputed.

^b^The Chi-square probability level can vary between low (0.04) and high (0.31) values for different imputed data sets.

### Right-skewed distribution and a 1% dose tail

The top 1% of the cumulative dose distribution showed unusually high values associated with doses from heavily-trafficked intersections, as shown in Figure 3, where probability plots are presented with and without the intersection dose component included in total dose. Without intersection dose included, the probability plot of log dose in Figure 3, with the exception of one point, trends smoothly below the normal line at the high end. However, with intersection dose included there is an abrupt deviation, with points falling well outside the upper 95% confidence limit on the normal curve shown in the Figure. The same skewed distribution appears when plotting doses computed for other assumptions about the biological effectiveness factor, *B*, with the exception of the pure promoter model, *B*_4_ (results not shown). The skewed distribution at high doses was maintained no matter the limitation placed on imputation percentage (PDI) in 15 imputed data sets (results not shown).

## Discussion

The past decade has seen an increasing understanding of the importance of the impact on chronic disease risk of exposures at all stages of life, especially exposures occurring during the early years of life when many organs are still undergoing rapid and formative growth [17]. Because many epidemiological studies of chronic illnesses such as cancer are usually initiated when the study population have already reached adulthood, capturing decades-old data on critical factors such as nutrition, exercise, and environmental exposures is a major challenge.

Residential address histories can be gathered by interview with relative ease, and we previously developed models for using histories obtained directly from patient interviews to estimate residential exposure to PAHs from traffic sources [35,36] which we then calibrated against environmental measurements for participants in a breast cancer case–control study [18]. We further refined our methodology and historical database to take into account long-term changes in emission control technology [21]. A primary limitation, however, continues to be the completeness and accuracy of patient address recall. The present work provides a methodology to address this type of limitation by applying multiple imputation methods to extract the maximum information from available data, making it possible to use incompletely known residential data to put credible bounds on exposure metrics.

The early (pre-1960) surrogate dosage for the breast cancer study subjects averaged 50% of the post-1960 dose, showing that it can make a substantial contribution to lifetime dosage.^b^ Interestingly, because this surrogate prior to scaling does not depend on information obtained from interview, it cannot directly introduce any recall bias into the dose estimates, although some bias can be carried over to it from the post-1960 averages. The way the early surrogate is defined in our example, without separate standardization by geographic region, the only connection to an individual is through age. However, the weak association (p = 0.13) between PDI and case status in our breast cancer data implies possible differential bias due to missing residential data; this will be investigated in a forthcoming epidemiological analysis that uses the present method to predict breast cancer risk.

Our finding of a consistent, strongly right-skewed distribution of traffic doses, associated with heavily trafficked intersections, means that non-standard distributions of exposure must be anticipated in studying traffic emissions. And, for our example data, this high-dose subpopulation is worthy of attention in future risk analyses that include these study subjects.

Because Pearson correlation coefficients between dose calculated with different biological effectiveness factors ranged from 0.33 to 0.79 with no limits set on PDI (PDI < = 100), the sensitivity of risk to age at exposure could be important in determining the strength of an association with health outcome; moreover, it suggests that regressions against health data might shed some light on which biologic model is most appropriate.

## Conclusions

In historical reconstructions of exposure, investigators typically identify data that have been collected for environmental monitoring and not for epidemiology, and that do not fully cover the population of interest. We suggest here a way of filling in missing data that we hope will spur increased use of historical exposure reconstruction in health studies. A novel feature of our exposure reconstruction approach is an incompleteness index (PDI) that can be used to study the degree to which imputation might create artifactual dosages and, subsequently, improper associations with health outcomes -- a form of differential bias to which researchers need to be sensitive in general [37,38] and specifically in traffic air pollution studies [39].

## Endnotes

^a^The risk equation in the main text, Eq. 1, was considered to be linear in dose. If the log odds is considered to be non-linear in exposure, then there would be terms in powers of Z as well a term in the log of Z [23,40,41] and possibly powers of log of Z. We did not consider such an extension of the methodology in the examples given in this paper.

^b^One way to reduce the relative contribution of the early surrogate would be to trade one approximation for another, namely running the full exposure model at times earlier than the period for which full emissions and traffic data exists. This extrapolation could introduce misclassification due to errors in traffic flow rates and tailpipe emission rates, but might introduce less misclassification than would result from relying on an early surrogate. Depending on the variability in a model, an analyst might explore extrapolating the individualized model back in time to reduce the contribution from the early dose surrogate to a modest level relative to total dose.

## Abbreviations

A: Constant term in regression, including covariate contributions to risk; B1 through B4: Functional forms for biological effectiveness; B(y’-yby-yb, i): Biological effectiveness factor in subject, *i*, that relates exposure at a particular biological age, *y*’-*y*_b_, to detection of disease at a later biological age, *y*-*y*_b_; BaP: Benzo(a)pyrene; β: Vector of regression coefficients for *Z*(*i*,*y*); D(y’ri[y′]): Transfer function; E(y′i): Exposure at date, *y′*, for subject, *i*; ϵ(y′): Emission from a vehicle tailpipe in year, *y′*; pi: Probability of an individual getting a disease; PDI: Percentage of dose imputed; ri[y′]: Residence of a subject at date, *y′*; T(y′): Average number of vehicles on an average road on date *y′*; V1(i)V2(i): Random variables with mean of unity; Y: Date of disease detection; y′: Date of an exposure; yb: Date of birth; ystop: Last exposure date of interest; Z(i,y): Vector of effective doses for individual, *i*, in year, *y*; Zcalc: Modeled component of individual dose; Zimp: Imputed part of dose.

## Competing interests

The authors declare that they have no competing interests.

## Authors’ contributions

JB developed and computed the exposure model; he drafted the manuscript. SS participated in the design and coordination of the approach and helped to draft the manuscript. ST managed study subjects, their interviews, and measurements. IM tested the multiple imputations for utility in regressions with health outcomes, as well as analyzed statistical properties of the exposure distributions. MG conceived of the LIBCSP and the need for a geographic model to assess airborne PAH exposure, managed study subjects, their interviews, and measurements; she promoted the use of multiple imputation and helped to draft the manuscript. All authors read and approved the final manuscript.

## Supplementary Material

Additional file 1: Figure S1-1 Percentage of addresses successfully geocoded by year of arrival at residence; **Figure S1-2.** Vehicle registrations in New York State by year; **Figure S1-3.** Segments of roads within 100 meters of major intersections where PAH emissions are increased in dispersion model; **Figure S1-4.** Early surrogate (dose before 1960) versus age; **Table S1-1.** Imputation by interpolation: Pearson correlation coefficients between doses computed with different functional forms for the biologic effectiveness factor; **Table S1-2.** Imputation by interpolation: Spearman correlation coefficients between doses computed with different functional forms for the biologic effectiveness factor. Click here for file

Additional file 2 **Details about imputation of the transfer function, *D, *using the method of imputation by place. Table S2-1.** Variables imputed and/or used in imputation; Comparison of historical dose quartiles with exposure quartiles at most recent address by dose categories. **Table S2-2.** Chi-Square Table comparing end-of-period (1995) dose to cumulative dose; Issue of multiple comparisons; Additional details of the LIBCSP meteorological dispersion model; **Table S2-3.** Default meteorological dispersion model. Click here for file
