# Supplementary material for: Imputation method for lifetime exposure assessment in air pollution epidemiologic studies
Source: Environ Health. 2013 Aug 7;12:62. doi: 10.1186/1476-069X-12-62 (PMC3751034; doi:10.1186/1476-069X-12-62)
Supplement: Additional file 1: Figure S1-1 — Percentage of addresses successfully geocoded by year of arrival at residence; Figure S1-2. Vehicle registrations in New York State by year; Figure S1-3. Segments of roads within 100 meters of major intersections where PAH emissions are increased in dispersion model; Figure S1-4. Early surrogate (dose before 1960) versus age; Table S1-1. Imputation by interpolation: Pearson correlation coefficients between doses computed with different functional forms for the biologic effectiveness factor; Table S1-2. Imputation by interpolation: Spearman correlation coefficients between doses computed with different functional forms for the biologic effectiveness factor. [file 1476-069X-12-62-S1.doc]

Additional File 1 for:

"Imputation method for lifetime exposure assessment in air pollution epidemiologic studies"

Jan Beyea, Steven D. Stellman, Susan Teitelbaum, Irina Mordukhovich, Marilie D. Gammon

Table of contents:

.

Figure S1-1. Percentage of addresses successfully geocoded by year of arrival at residence.

Figure S1-2. Vehicle registrations in New York State by year.

Figure S1-3. Segments of roads within 100 meters of major intersections.

Figure S1-4. Early surrogate (dose before 1960) versus age.

Text: Explanation of format used in Tables S1 and S2.

Table S1-1. Imputation by interpolation: Pearson correlation coefficients between cumulative traffic dose 1960-1990 and other dose surrogates calculated for one imputation

Table S1-2. Imputation by interpolation: Spearman correlation coefficients between cumulative traffic dose 1960-1990 and other dose surrogates calculated for one imputation


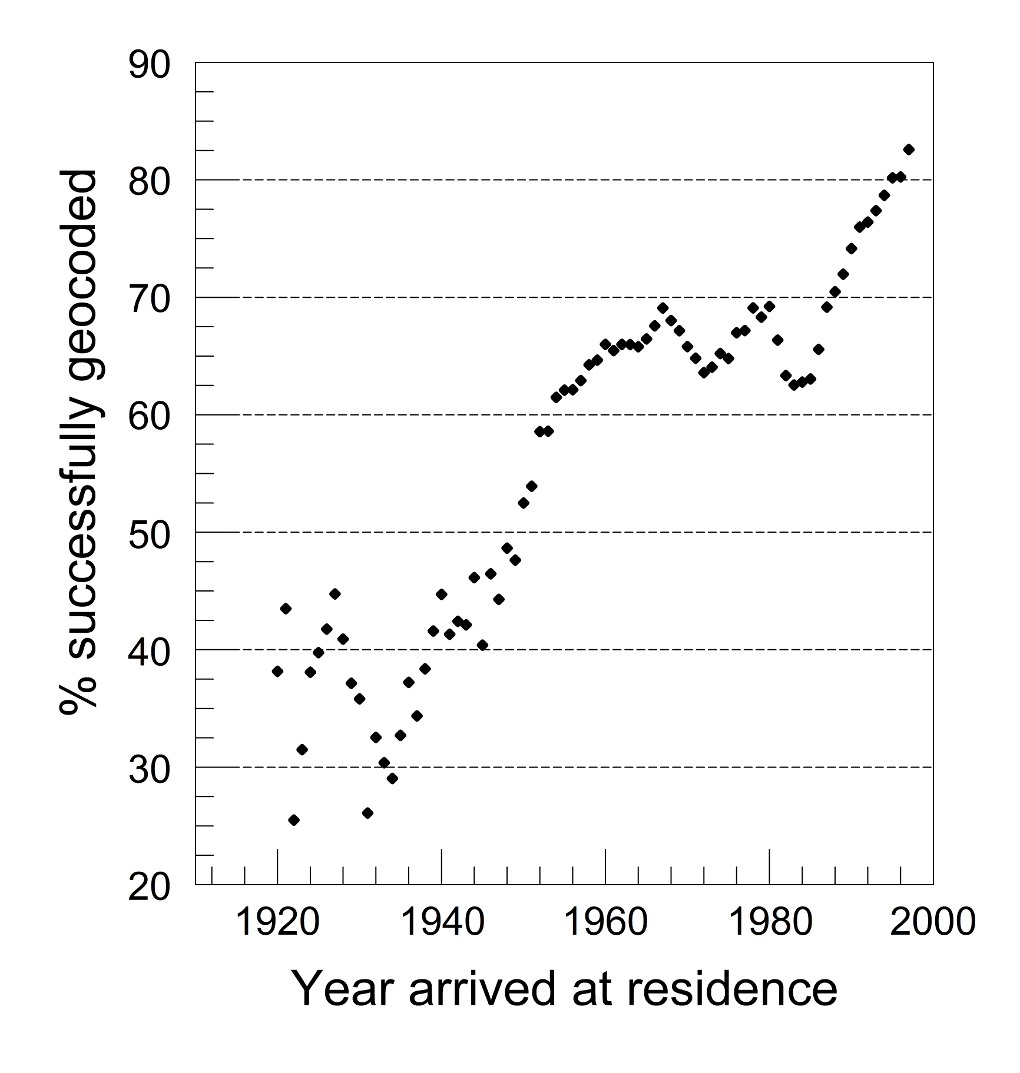


Additional File 1, Figure S1-1. Percentage of addresses successfully geocoded by year of arrival at residence. Long Island Breast Cancer Study Project, 1996-1997 [1].

Additional File 1, Figure S1-2. Vehicle registrations in New York State by year. Long Island Breast Cancer Study Project, 1996-1997 [1].


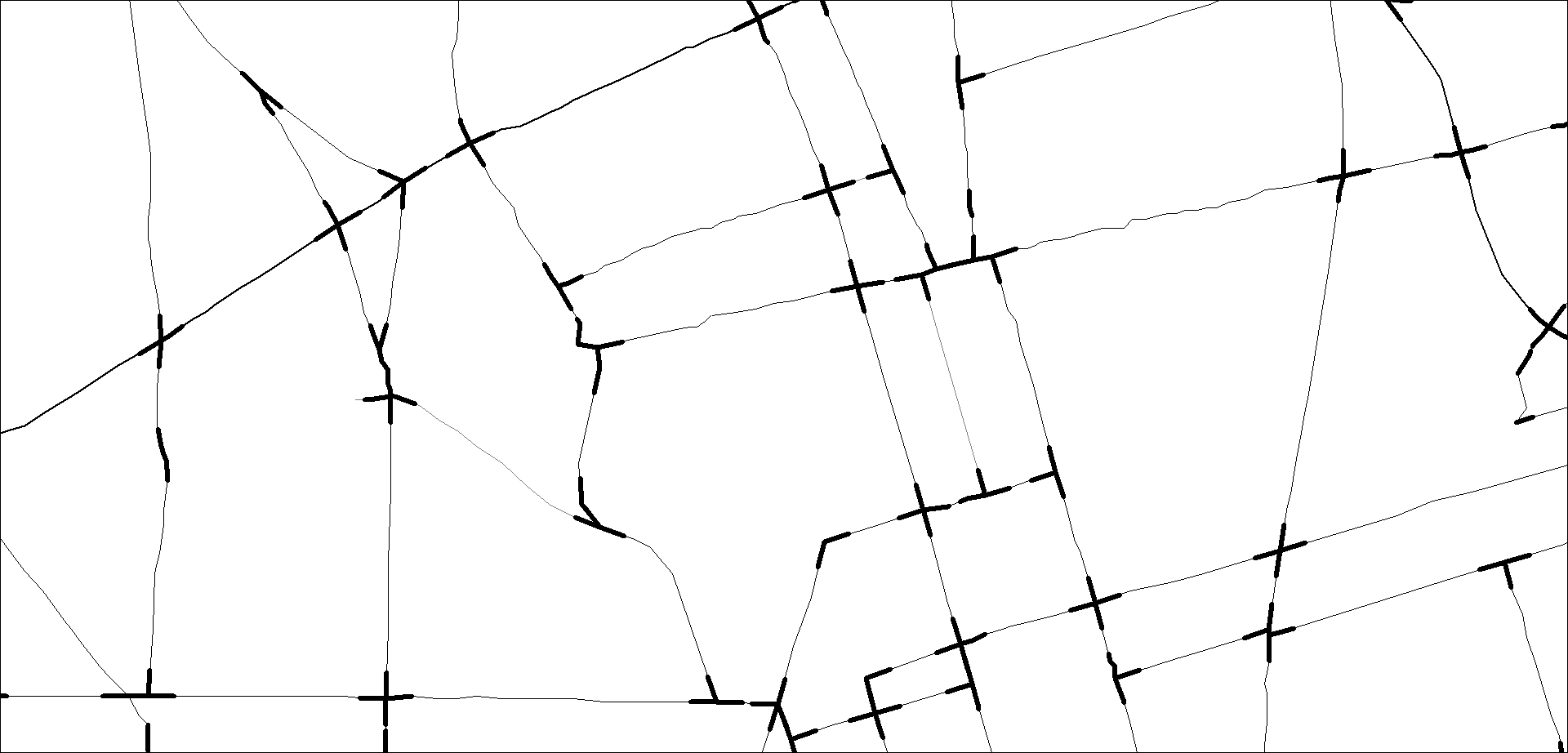


Additional File 1, Figure S1-3a. Segments of roads within 100 meters of major intersections where PAH emissions are increased in dispersion model. Shown as cross-like structures, darkened for visual emphasis, within a 4-km by 6-km wide map selection from Nassau County, Long Island, New York. See map below (Figure S1-3b) for location of the selection on Long Island. Long Island Breast Cancer Study Project, 1996-1997 [1].


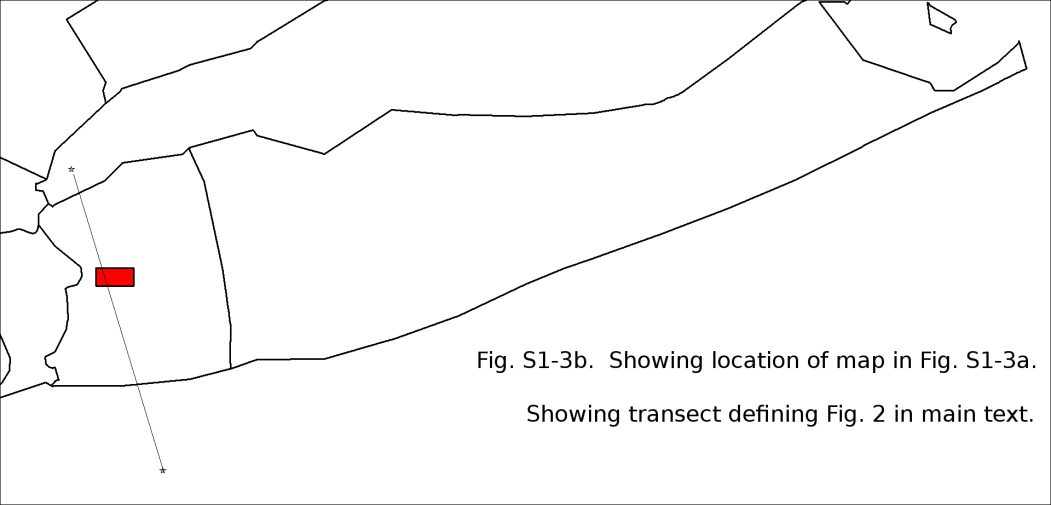


Additional File S1, Figure S1-4. Early surrogate (dose before 1960) versus age. Solid curve is a LOESS fit (locally weighted regression line). N = 3064. Mean dose = 96. Mmean age = 58. Relative dose units: 1 unit = 1 year's average dose in 1995. Long Island Breast Cancer Study Project, 1996-1997 [1]

Additional File 1, Tables S1-1 and S1-2 (shown below) present correlation coefficients, both Pearson and Spearman, between doses computed for different biologic effectiveness factors. In contrast to Table 1 of the main text, the doses used in Tables S1-1 and S1-2 were computed using the method of "imputation by interpolation" for the transfer function, as compared to the method of "imputation by Place." Results are presented for a range of limits on the percentage of dose imputed (PDI), which is useful in assessing the robustness of the imputations. The limit on allowed PDI is varied in six steps across the Table, beginning with zero, i.e., complete case analysis, and ending with no limitation on the amount of imputation (PDI <= 100%).

| Additional File 1, Table S1-1. Imputation by interpolation: Pearson correlation coefficients between doses computed with different functional forms for the biologic effectiveness factor for one imputation | | | | | | | |
| --- | --- | --- | --- | --- | --- | --- | --- |
|  |  | Samples of women limited by allowed percentage of imputed dose | | | | | |
| Dose surrogate |  | CCA (0%) | PDI < 20% | PDI < 40% | PDI < 60% | PDI < 80% | PDI < 100% |
|  | N: | 559 | 1122 | 1449 | 1729 | 2022 | 2869 |
|  |  |  |  |  |  |  |  |
| Cumulative dose 1960-1990 (comparison dose variable) |  | (comparison dose variable) | | | | | |
| Dose for 1995 only (Promoter model) |  | 0.58 | 0.39 | 0.40 | 0.40 | 0.42 | 0.38 |
| Peak annual dose in 1960-1990 period (Threshold model) |  | 0.99 | 0.93 | 0.93 | 0.92 | 0.91 | 0.75 |
| Cumulative dose X (onset age)-2 (Age sensitive model)a, b |  | 0.88 | 0.87 | 0.85 | 0.84 | 0.82 | 0.80 |
| Pre-1960surrogate (V_1_*Z_1_) |  | -0.049 | -0.018 | -0.018 | -0.0075 | -0.0078 | -0.0080 |
| a) Weighting based on mathematical fit to radiation risks of excess breast cancer in atomic bomb survivors.  b) Onset age was approximated by the midpoint of each 4 or 5 year age group in the Long Island Breast Cancer Study Project study population. | | | | | | | |

| Additional File 2, Table S1-2. Imputation by interpolation: Spearman correlation coefficients between doses computed with different functional forms for the biologic effectiveness factor for one imputation. | | | | | | | |
| --- | --- | --- | --- | --- | --- | --- | --- |
|  |  | Samples of women limited by allowed percentage of imputed dose | | | | | |
| Dose surrogate |  | CCA (0%) | PDI < 20% | PDI < 40% | PDI < 60% | PDI < 80% | PDI < 100% |
|  | N: | 559 | 1122 | 1449 | 1729 | 2022 | 2869 |
|  |  |  |  |  |  |  |  |
| Cumulative dose 1960-1990 (comparison dose variable) |  | (comparison dose variable) | | | | | |
| Dose for 1995 only (Promoter model) |  | 0.84 | 0.77 | 0.72 | 0.70 | 0.67 | 0.58 |
| Peak annual dose in 1960-1990 period (Threshold model) |  | 0.98 | 0.95 | 0.94 | 0.93 | 0.89 | 0.59 |
| Cumulative dose X (onset age)-2 (Age sensitive model)a, b |  | 0.81 | 0.73 | 0.73 | 0.74 | 0.75 | 0.75 |
| Pre-1960surrogate (V_1_*Z_1_) |  | 0.011 | 0.032 | 0.0022 | 0.023 | 0.016 | -0.0030 |
| a) Weighting based on mathematical fit to radiation risks of excess breast cancer in atomic bomb survivors.  b) Onset age was approximated by the midpoint of each 4 or 5 year age group in the Long Island Breast Cancer Study Project study population. | | | | | | | |

Reference:

1. Gammon MD, Neugut AI, Santella RM, Teitelbaum SL, Britton JA, Terry MB, Eng SM, Wolff MS, Stellman SD, Kabat GC *et al*: **The Long Island Breast Cancer Study Project: Description of a multi-institutional collaboration to identify environmental risk factors for breast cancer**. *Breast Cancer Res Treat* 2002, **74**(3):235-254.
