# Supplementary material for: Imputation method for lifetime exposure assessment in air pollution epidemiologic studies
Source: Environ Health. 2013 Aug 7;12:62. doi: 10.1186/1476-069X-12-62 (PMC3751034; doi:10.1186/1476-069X-12-62)
Supplement: Additional file 2 — Details about imputation of the transfer function, D, using the method of imputation by place. Table S2-1. Variables imputed and/or used in imputation; Comparison of historical dose quartiles with exposure quartiles at most recent address by dose categories. Table S2-2. Chi-Square Table comparing end-of-period (1995) dose to cumulative dose; Issue of multiple comparisons; Additional details of the LIBCSP meteorological dispersion model; Table S2-3. Default meteorological dispersion model. [file 1476-069X-12-62-S2.doc]

Additional File 2 for,

"Imputation method for lifetime exposure assessment in air pollution epidemiologic studies."

Jan Beyea*,^1^ Steven D. Stellman.^2^

1: Consulting in the Public Interest (CIPI), 53 Clinton Street, Lambertville, NJ; 2: Department of

Epidemiology, Mailman School of Public Health, Columbia University, NY, NY.

* jbeyea@cipi.com.

Table of Contents

Details about imputation of the transfer function, *D*, using the method of imputation by place.

Comparison of historical dose quartiles with exposure quartiles at most recent address by dose categories.

Multiple Comparisons of dose models.

Some limitations of the exposure modeling used for the numerical examples.

**Additional details about imputation of the transfer function, *D*, using the method of Imputation by place.**

As stated in the main text when discussing the "Breast cancer example," we noted that there were a total of 8,319 locations in Nassau and Suffolk counties divided among the 3,064 women in the study. Address coordinates at the street level, and hence *D*-values, were identifiable for 5,383 of these locations. The remaining *D*-values (~ 3,000) are considered "unobserved" and had to be imputed. The unobserved *D*-values were imputed in the time period after 1960, the first year when full information existed on traffic flows and tailpipe emissions. A woman's total dose (opportunity) depended on multiple *D*'s, if she lived in more than one location during the study period. Dose, besides depending on *D*-values, also depended on the relative duration lived at each residence and the historical sequence of residence relative to emissions decline.

Rather than impute *D*’s directly when using the method of imputation by place, we worked with *D*-values normalized to the average of observed values, with Place average computed separately for cases and controls. The final *D*-values were then obtained by multiplying the appropriate Place average by the imputed *D*_norm_. We made this choice for several reasons. First, the normalized *D*-values (*D*_norm_) were uncorrelated with breast cancer status (p > 0.9). Second, among the known *D*-values we found a long tail in the distribution with a 1%-frequency rate that would not show up well when restricted to individual Places, which have counts ranging from a few to sixty for control women. Thus, the data itself could not tell us how the long-tailed distribution varied with Place. Based on previous work [1], we expected abnormally high relative values to occur in any census Place near intersections, so we assumed that the long-tail would occur everywhere. Our choice of imputing *D*_norm_’s, in effect, assigns to each Place the global, non-standard distribution with its long tail.

Multiple imputation of the missing transfer function along with any missing covariate values, was carried out using fully conditional specification as implemented in the R-program, “Multivariate Imputation by Chained Equations (MICE)” [2]. Because of the observed long tail in the dose-distribution that could not be fit with a log-normal or other standard distribution, we chose the option for predictive mean matching in the MICE software, which insures that imputed *D*_norm_’s come from “observed" values. The predictive mean matching method ensures that imputed values are plausible and is more appropriate than the regression method if the normality assumption is violated [3].

Imputation was carried out in two steps. The first step accounted for women who had more than one residence with a missing *D*_norm_ and who also had a value missing for one of the covariates listed in Table S2-1. Only one of these women's residences with missing *D*_norm_'s was included in the first imputation. This established imputed values of missing covariates, which could then be used in the second imputation of the residual *D*_norm_'s not imputed in the first round. As a result, a woman who happened to be missing a covariate, say, highest level of education achieved, was assigned the same value of education level for every residence. That value might change between imputed data sets, but it would be the same for each of her residences within an imputed data set.

| Additional File 2, Table S2-1. Variables imputed and/or used in imputation^a^ | | |
| --- | --- | --- |
| Imputation  Round | Direct variables | Interactions with *D*_norm_ |
| Round 1b | *D*_norm_  Active smoking status (never, past, current)  Highest level of education achieved  Fertility problems  History of benign breast disease  Family history of breast cancer  Combined estrogen receptor and progesterone receptor status among cases  Parity  Nulliparity  BMI at reference date  Dietary intake of PAH [4] Lifetime intake of grilled or barbecued and smoked meats [4] | Active smoking status (never, past, current)  Passive smoking status (never, past, current)  Menopausal status  Nulliparity  Dietary PAH variables  Combined estrogen receptor and progesterone receptor status among cases |
| Round 2c | *D*_norm_s not imputed in Round 1  All covariates from Round 1 (now with all missing values imputed)  Lifetime alcohol intake [5]  Case-control status  Age at diagnosis  Age at menarche  Total household income before taxes in last year  Centered age at first birth  Use of hormone replacement therapy  Use of oral contraceptives  Latina  Religion in which subject was raised  Total hours worked between 1960 and 1990 |  |
| a) Covariates obtained through interview are described in Gammon et al. [6], unless another citation is indicated.  b) Missing *D*_norm_’s and any missing covariates jointly imputed, using observed values of all of them.  c) A woman’s second or later missing *D*_norm_ was imputed in Round 2. These residual *D*_norm_’s were imputed using covariates from Round 1 along with the new covariates added in Round 2, which were included, despite their low correlation with the *D*_norm_'s, to insure that any of their missing values were filled in with imputed values. | | |

We performed linear multivariate regression on *D*norm’s, to decide which covariates to use in conditional specifications involving *D*norm’s, retaining variables for which p-values were less than 0.25. These variables are listed in Table S2-1 under the Round-1 imputations. Any missing values of these covariates were also imputed in the process. Also included were biologically interesting interaction terms with *D*norm’s, which are listed in the second column of Table S2-1. By including interaction terms in the imputation, we carry over correlations found in the observed data to the unobserved data needing imputation

After imputing the *D*norm’s with these variables, the covariates not included in the first round (listed in Table S2-1, Round 2) had any missing values imputed by MICE as part of the second imputation. Fifteen imputations were carried out for our numerical examples. Five are considered sufficient for combining confidence limits across multiple imputations using Rubin’s rules [7].

**Comparison of historical dose quartiles with exposure quartiles at most recent address by dose categories.**

In the absence of methods to deal with historical exposure data, few epidemiologic studies to date have attempted to gather complete lifetime residential addresses, and consequently many studies base exposure estimates on current or recent residences as proxies for lifetime histories [8]. This must clearly result in misclassification of exposure. To get some idea of the extent of such misclassification, we calculated Pearson correlation coefficients between cumulative and current doses as shown in Table 1 of the main text. This Table indicated how these different dose estimates matched up when considered as continuous variables (r–values below 0.6). Table S2-2 below shows how cumulative and current doses match up using a categorical approach in a frequency Table. Specifically, Table S2-2 compares dose quartiles for the last year of the study period to the estimated quartiles for dose from 1960 to 1990. The results are for a typical imputation. Table S2-2 indicates that the quartile assignments for recent dose are modestly consistent with the quartile assignments for cumulative doses. 43% to 61% of women were classified the same under the two measures depending on the quartile number. Still, there is significant misclassification, which could lead to an attenuation of any effect between dose and disease risk, were the wrong measure to be chosen for analysis. We found that the average distance of a move on Long Island for our study subjects was less than 10 km, which suggests that, aside from intersection complications, study subjects remained in generally the same area along the 120-km long study area. Because considerable dose gradient occurs along the Island study area [1], the tendency to remain relatively close to previous residences may help explain the general consistency of the two exposure measures.

| Additional File 2, Table S2-2. Chi-Square Table comparing end-of-period (1995) dose to cumulative dosea) | | | | |
| --- | --- | --- | --- | --- |
|  | Quartile of 1995 dose | | | |
|  | 1 | 2 | 3 | 4 |
| Quartile of dose 1960-1990 |  |  |  |  |
| 1 | 375 | 198 | 71 | 51 |
| 2 | 154 | 302 | 180 | 72 |
| 3 | 96 | 113 | 328 | 150 |
| 4 | 73 | 86 | 120 | 426 |
|  |  |  |  |  |
| Total of column | 698 | 699 | 699 | 699 |
| a) For one imputation. The number of women in the Table is less than the number for which we have total dose, because of the difficulty in imputing 1995 doses for women for whom we have only pre-arrival dose. | | | | |

Alternate transfer function for out-of-area dose surrogates.

The choice of transfer function used in the out-of-area surrogate was based on an average over study subjects with calculated transfer function. For sensitivity analysis, it is possible to take a different approach, namely to adjust the average transfer function up or down to insure that there is no dependence of total average dose on year of arrival. This alternate approach produced only minor differences in total doses. Correlation coefficients were in the 99% range.

**Multiple Comparisons of dose models**:

We have considered a number of models for biological effectiveness of traffic pollution, because it is not clear that a simple exposure sum is the best metric for assessing health risk. How many of these models should be used in regressions against health data? There are few empirical data on the nature of the dose-response relation for traffic pollutants and the assumption of a simple dose-response relation implicit in the use of cumulative exposures seems a reasonable baseline strategy. Consideration of peak exposure also seems worthwhile, given the possibility of a non-linear dose response indicated in the earlier Long Island PAH-DNA adduct study of breast cancer [9] and the finding of a high dose cohort among the top 1% of cases in this study. The strong age effects found in radiation epidemiology for breast cancer suggest it is worth trying functional forms that vary strongly with age at exposure and/or age of onset, recognizing that chemical pollutants may have more complex interactions with cells than does ionizing radiation, due, for instance, to hormonal influences that might supplement DNA-damage. Still, the radiation functional forms will help in comparing and contrasting different possible dose-response functions.

Of course, when considering many functional forms and alternatives in regressions against health risk, there will be multiple comparisons made that could increase the chance of a false finding. However, at the current state of knowledge we do not want to miss an exposure-disease association [10, 11], so it is appropriate to work with a large set of dose measures.

**Additional details of the LIBCSP meteorological dispersion model used in the numerical examples**.

| Addition File 2, Table S2-3. Default meteorological dispersion model | | | |
| --- | --- | --- | --- |
| Quantity of interest | Data source | Comment | Options for sensitivity analysis |
| < 100 m of road | Chock dispersion model [12].^a)^ | Models merged at 100 m.^b)^ |  |
| > 100 m of road | Gaussian plume model. | Briggs dispersion parameters used. [13].^b)^ | Rural dispersion parameters |
| Meteorological data for wind speed and direction. | 1993 values from Brookhaven National Labs meterological station [14]ti |  | 1990 BNL values; 1990 and 1993 data from MacArthur airport [15]. |
| Mixing layer | 1993 values from [16].^c)^ |  | 1990 values. |
| Rain washout | Hourly mm of rainfall [14]. | Took standard function of rainfall rate [17]. | Scale factor |
| Photo-decay | Hourly pyranometer readings from [14]. | Proportional to the amount of sunlight, adjusted to give an average 6-hour decay rate.^d)^ | Scale factor. 1990 pyranometer readings. |
| Deposition velocity | [18] | Default value = 0.003 m/s | Scale factor |
| 1. Computationally, the Chock model was converted to a gaussian plume formulation by fitting the Chock predictions to spatially dependent, gaussian plume dispersion parameters applied to puffs emitted along the roadway. 2. At 100 meters from the road, the puffs were allowed to continue to expand using standard dispersion parameters, whose values for the default model were taken from 1993 Brookhaven Laboratory measurements (BNL). Values measured at Brookhaven in 1990 and values derived using PCRammet from data collected at MacArthur airport in 1990 and 1993 were used for sensitivity studies [19], [15]. Both Brookhaven Laboratory and MacArthur airport (Islip) are in Long Island. 3. Raw data converted to hourly mixing heights using the program, PCRammet [19]. 4. The default value was chosen to produce a mean BaP lifetime during daylight of 6 hours, a value within the wide range given in the literature. [20], [21]. | | | |

**Switching between different meteorological models at 100 meters**. Within 100 meters of a road segment, the Chock highway line model was used [12]. The model gives vertical and horizontal dispersion parameters as a function of stability conditions, wind speed, angle from the road, and distance. At 100 meters, we matched the values of the Chock dispersion parameters to comparable dispersion parameters in a Gaussian plume model, using a virtual source method [22]. The virtual source method adds a virtual distance to the roadway position sufficient to produce the desired plume width at 100 meters, assuming that the dispersion had been Gaussian from the beginning. The virtual distance is computed for both horizontal and vertical widths and may differ in each case. Armed with the matching Gaussian dispersion parameters, the Gaussian model can be used to follow the puff beyond 100 meters.

**Background BaP blown into the study area from outside it**. Previous traffic models have estimated regional background in a number of ways. For instance, they have used gasoline sales data to estimate background emissions, have used a constant value as a surrogate for the cumulative contribution of distant roads, or have estimated regional background from trends at rural monitoring stations and from national vehicle emissions. [23-25]. Although we have attempted to directly account for traffic emissions within 80 km of our study area, there are more distant roads, as well as other sources of PAH emissions, making it prudent to include a background term in the model. We provided two options for use in model calibration exercises [1]. In the first, a constant term was added to the on-island model prediction. For the second option, we allowed background to be proportional to the exposure calculated from the outer counties (all but Nassau, Suffolk, and Queens counties) out to 80 km from Long Island. In both approaches, the relative scale of the background term can be set by calibration to environmental measurements. The second option provided a better fit to the soil data used for model calibration, presumably because it captured some of the gradient caused by dispersion from the New York City Metropolitan region.

**Validation of the model**. As discussed in the section on the meteorological model in the main text, the meteorological dispersion model was validated against soil data collected as part of the project [1]. A meteorological dispersion model can predict soil concentrations because deposits from the air are proportional, on average, to airborne concentrations. The model was also tested against hourly CO-levels at a CO-monitoring station and against PAH-DNA adduct levels measured in the blood of study subjects and found to be consistent in both cases [1]. The model predictions for deposition to carpets, however, were not consistent with measurements of PAH levels found in dust vacuumed from the carpets of study subjects [1], but this no longer surprises us, because we have since learned that PAH in house dust has been found to not correlate with airborne concentrations [26], indicating that track-in, cooking splash, or other sources must dominate the concentration in carpet dust.

**Some limitations of the exposure modeling used for numerical examples:**

The major strengths of the model are that it incorporates higher relative emissions from intersections and that it has been validated and calibrated against soil data collected at 500+ residences and checked against other environmental data. However, there are some limitations, which we now discuss, that will lead to some exposure misclassification.

Sea and Shore breezes. For several hours each day, sea and shore breezes affect the mixing layer across the island. Although their impact on wind directions was included in the model through use of Long Island wind data, the impact on mixing layer was not fully incorporated, because only average mixing layers were utilized as a cap on vertical expansion of puffs. This means that we explicitly neglected the “thermal internal boundary layer” (TIBL) [27] that forms over the Island for several hours when the wind is blowing from the ocean on warm days. Neglect of the TIBL is a limitation of the model applicable to a segment of each warm day. It could have led to some exposure misclassification.

Exposure while traveling in vehicles. We did not attempt to account for exposure to traffic PAH while in a moving vehicle [28], [29], because no questions directly relevant to in-vehicle modeling were included in the study questionnaire. We expected this limitation to lead to some misclassification for women residing far from major roads, based on order- of-magnitude calculations. Recent work in Southern California has suggested that this could be an important limitation for many subjects, at least in some locations [30]. As a result, researchers carrying out future studies of this type might want to gather information at interview about historical travel patterns.

Sources of PAH not modeled. The primary goal of the model used for our numerical examples was to obtain estimates of life course vehicular traffic exposure. Thus, for this model, we have purposely not focused on other sources of PAH, which were assessed as part of the LIBCSP. For example, as stated in the introduction to the main text, we have available individualized estimates for PAH in diet and exposure to passive/active smoking. Lifetime intake of grilled/smoked meat [31], and long-term passive smoking—but not current or former active smoking [32]—have been associated with breast cancer in our study population. We also have data from interview on individual use of both wood stoves and fireplaces as a categorical variable, but they are not needed as an example of our approach to life course exposure assessment. Within the category of PAH air exposure, as stated in the introduction to the main text, we focused in this paper on vehicular traffic because it is a major source of both indoor and outdoor exposures to PAH, and often the largest source in areas near cities, as has been confirmed in a number of experimental studies [33-38].

The fact that our modeled distribution of doses has a long tail due to traffic intersections is an advantage in our case, because for subjects in the tail their traffic exposure is likely to rise well above contributions from other PAH sources. Furthermore, neither emissions from residential heating systems nor emissions from industry nor emissions from electric power sources are likely to be correlated with intersections. In general, a non-standard distribution with a long tail attributable to a unique set of exposure circumstances, despite the complexity that may arise in the statistical analysis, may turn out to be quite useful, because it may prevent confounding occurring throughout the entire study population.

Exposure opportunity. Residential exposures in our geographic model were calculated assuming a woman was always present at her residence. As a surrogate for work exposure, we have estimates from interview of total hours worked at each job over a study subject's lifetime, which can be used in regressions against health data to control for work exposures.

References.

1. Beyea J, Hatch M, Stellman SD, Santella RM, Teitelbaum SL, Prokopczyk B, Camann D, Gammon MD: **Validation and calibration of a model used to reconstruct historical exposure to polycyclic aromatic hydrocarbons for use in epidemiologic studies**. *Environ Health Perspect* 2006, **114**(7):1053-1058.

2. van Buuren S, Groothuis-Oudshoorn K: **Multivariate Imputation by Chained Equations in R**. *J Stat Software* 2011, **45**(3):1-67.

3. Horton NJ, Lipsitz SR: **Multiple Imputation in Practice: Comparison of Software Packages for Regression Models with Missing Variables**. *The American Statistician* 2001, **55**(3):244-254.

4. Shantakumar S, Gammon MD, Eng SM, Sagiv SK, Gaudet MM, Teitelbaum SL, Britton JA, Terry MB, Paykin A, Young TL *et al*: **Residential environmental exposures and other characteristics associated with detectable PAH-DNA adducts in peripheral mononuclear cells in a population-based sample of adult females**. *J Expo Anal Environ Epidemiol* 2005, **15**(6):482-490.

5. Terry MB, Zhang FF, Kabat G, Britton JA, Teitelbaum SL, Neugut AI, Gammon MD: **Lifetime alcohol intake and breast cancer risk**. *Ann Epidemiol* 2006, **16**(3):230-240.

6. Gammon MD, Neugut AI, Santella RM, Teitelbaum SL, Britton JA, Terry MB, Eng SM, Wolff MS, Stellman SD, Kabat GC *et al*: **The Long Island Breast Cancer Study Project: Description of a multi-institutional collaboration to identify environmental risk factors for breast cancer**. *Breast Cancer Res Treat* 2002, **74**(3):235-254.

7. Li KH, Raghunathan TE, Rubin DB: **Large-Sample Significance Levels from Multiply Imputed Data Using Moment-Based Statistics and an F Reference Distribution**. *J Am Stat Assoc* 1991, **86**(416):1065-1073.

8. McElroy JA, Remington PL, Trentham-Dietz A, Robert SA, Newcomb PA: **Geocoding addresses from a large population-based study: lessons learned**. *Epidemiology* 2003, **14**(4):399-407.

9. Gammon M, Sagiv S, Eng S, Shantakumar S, Gaudet M, Teitelbaum S, Britton J, Terry M, Wang L, Wang Q *et al*: **Polycyclic aromatic hydrocarbon-DNA adducts and breast cancer: a pooled analysis.** *Arch Environ Health* 2004, **59**(12):640-649.

10. Blair A, Stewart PA: **Correlation between different measures of occupational exposure to formaldehyde**. *Am J Epidemiol* 1990, **131**(3):510-516.

11. London L, Myers J: **Use of a crop and job specific exposure matrix for retrospective assessment of long-term exposure in studies of chronic neurotoxic effects of agrichemicals**. *Occup Environ Med* 1998, **55**(3):194-201.

12. Chock DP: **A simple line-source model for dispersion near roadways**. *Atmos Environ* 1978, **12**:823-829.

13. Catalano JA, Turner BD, Novak JH: **User's Guide for RAM -- Second Edition**. In*.* Research Triangle Park: Environmental Protection Agency; 1987.

14. **Brookhaven National Laboratory Climatology Data Files, 1960-1993 http://www.bnl.gov/weather/**

15. **Hourly Surface Observations, Islip. http://www.ncdc.noaa.gov/**

16. **Mixing Heights, Islip and Atlantic City for 1990 and 1993. http://www.ncdc.noaa.gov/**

17. Ramsdell JV, Jr., Simonen CA, Burk KW: **Regional Atmosphere Transport Code for Hanford Emission Tracking (RATCHET)**. In*.* Richland, WA: Battelle Pacific Northwest Laboratories, PNWD-2224; 1994.

18. NCRP: **Uncertainty in NCRP Screening Models Relating to Atmospheric Transport, Deposition and Uptake by Humans**. In*.* Bethesda: National Council on Radiation Protection and Measurement; 1993.

19. USEPA: **PCRAMMET User's Guide**. In*.* Research Triangle Park: Environmental Protection Agency; 1999: 95.

20. Huang X-D, Dixon D, Greenberg B: **Increased PAH toxicity following their photomodification in natural sunlight: impacts on the Duckweed Lemma Gibba L. G-3.** *Ecotox Environ Safety* 1995, **32**:194-200.

21. Fan Z, Chen D, Birla P, Kamens RM: **Modeling of nitro-polycyclic aromatic hydrocarbon formation and decay in the atmosphere**. *Atmos Environ* 1995, **27A**(7):1139-1152.

22. Clegg AJ: **An on line atmospheric dispersion model (OLADMO) for the World Wide Web**. *Surv Geophys (2006) 27:433–485* 2006, **27**:433-485.

23. Viras LG, Siskos PA, Stephanou E: **Determination of polycyclic aromatic hydrocarbons in Athens atmosphere**. *Internat J Environ Anal Chem* 1987, **28**:71-85.

24. Raaschou-Nielsen O, Hertel O, Vignati E, Berkowicz R, Jensen SS, Larsen VB, Lohse C, Olsen JH: **An air pollution model for use in epidemiological studies: evaluation with measured levels of nitrogen dioxide and benzene**. *J Expo Anal Environ Epidemiol* 2000, **10**:4-14.

25. Raaschou-Nielsen O, Andersen Z, Hvidberg M, Jensen S, Ketzel M, Sorensen M, Hansen J, Loft S, Overvad K, Tjonneland A: **Air pollution from traffic and cancer incidence: a Danish cohort study**. *Environ Health* 2011, **10**(1):67.

26. Fromme H, Lahrz T, Piloty M, Gebhardt H, Oddoy A, Ruden H: **Polycyclic aromatic hydrocarbons inside and outside of apartments in an urban area**. *Sci Total Environ* 2004, **326**(1-3):143-149.

27. Luhar A, Sawford B: **An examination of existing shoreline fumiagtion models and formulation of an improved model.** *Atmos Environ* 1996, **30**:609-620.

28. Ott W, Switzer P, Willits N: **Carbon monoxide exposures inside an automobile traveling on an urban arterial highway**. *J Air Waste Manage Assoc* 1994, **44**(8):1010-1018.

29. Brice R, Roesler J: **The exposure to carbon monoxide of occupants of vehicles moving in heavy traffic**. *JAPCA* 1966, **16**(11):597-600.

30. Wu J, Tjoa T, Li L, Jaimes G, Delfino R: **Modeling personal particle-bound polycyclic aromatic hydrocarbon (pb-pah) exposure in human subjects in Southern California**. *Environ Health* 2012, **11**(1):47.

31. Steck SE, Gaudet MM, Eng SM, Britton JA, Teitelbaum SL, Neugut AI, Santella RM, Gammon MD: **Cooked meat and risk of breast cancer--lifetime versus recent dietary intake**. *Epidemiology* 2007, **18**(3):373-382.

32. Gammon MD, Eng SM, Teitelbaum SL, Britton JA, Kabat GC, Hatch M, Paykin AB, Neugut AI, Santella RM: **Environmental tobacco smoke and breast cancer incidence**. *Environ Res* 2004, **96**(2):176-185.

33. Dubowsky SD, Wallace LA, Buckley TJ: **The contribution of traffic to indoor concentrations of polycyclic aromatic hydrocarbons**. *J Expo Anal Environ Epidemiol* 1999, **9**(4):312-321.

34. Lim LH, Harrison RM, Harrad S: **The contribution of traffic to atmospheric concentrations of polycyclic aromatic hydrocarbons**. *Environ Sci Technol* 1999, **33**:3538-3542.

35. Harkov R, Greenberg A, Darack F, Daisey JM, Lioy PJ: **Summertime variations in polycyclic aromatic hydrocarbons at four sites in New Jersey**. *Environ Sci Technol* 1984, **18**:287-291.

36. Dickhut RM, Canuel EA, Gustafson KE, Liu K, Arzayus KM, Walker SE, Edgecombe G, Gaylor MO, MacDonald EH: **Automotive sources of carcinogenic polycyclic aromatic hydrocarbons associated with particulate matter in the Chesapeake Bay Region**. *Environ Sci Technol* 2000, **34**(21):4635-4640.

37. Dunbar JC, Lin CI, Vergucht I, Wong J, Duran JL: **Estimating the contributions of mobile sources of PAH to urban air using real-time PAH monitoring**. *Sci Total Environ* 2001, **279**(1-3):1-19.

38. Levy JI, Houseman EA, Spengler JD, Loh P, Ryan L: **Fine particulate matter and polycyclic aromatic hydrocarbon concentration patterns in Roxbury, Massachusetts: a community-based GIS analysis**. *Environ Health Perspect* 2001, **109**(4):341-347.
